# Flexural Strength of Modern CAD/CAM Restoratives After Artificial Aging

**DOI:** 10.3390/ma17215178

**Published:** 2024-10-24

**Authors:** Thomas Melc, Thomas Attin, Tan Fırat Eyüboğlu, Mutlu Özcan

**Affiliations:** 1 Clinic of Conservative and Preventive Dentistry, Center for Dental Medicine, University of Zurich, 8032 Zurich, Switzerland; thomas.melc@uzh.ch (T.M.); thomas.attin@zzm.uzh.ch (T.A.); 2 Department of Endodontics, Faculty of Dentistry, Istanbul Medipol University, 34083 Istanbul, Türkiye; tfeyuboglu@yahoo.com; 3 Clinic of Masticatory Disorders and Dental Biomaterials, Center for Dental Medicine, University of Zurich, 8032 Zurich, Switzerland

**Keywords:** aging, CAD/CAM, CAD/CAM materials, dental materials, lithium disilicate, prosthodontics

## Abstract

Before clinical trials are initiated, studying the mechanical performance of modern CAD/CAM restorative materials exposed to aging conditions would provide insights on their performance in service. This study evaluated the impact of thermomechanical aging on various resin composite, ceramic, hybrid, and nano-filled resin composite materials after two polymerization modes. Specimens (3 × 4 × 14 mm^3^) were fabricated using (*n* = 12 per group) a universal composite (Filtek Supreme XTE photo-polymerized for either 40 s or 120 s per layer), hybrid ceramics (BRILLIANT Crios, GC Cerasmart, Lava Ultimate, VITA ENAMIC), glass ceramics (IPS e.max CAD, VITA Suprinity PC, Straumann n!ce), or feldspar ceramics (VITABLOCS Mark II, GC Initial LRF). In each group, half of the specimens underwent thermomechanical aging. A three-point bending test was applied to all specimens and the results were statistically analyzed (α = 0.05). Glass ceramics and hybrid ceramics presented higher flexural strength values than feldspar ceramics and the universal composite before and after aging (*p* < 0.05). Thermomechanical cycling affected the flexural strength of all materials (*p* < 0.05) except Lava Ultimate, Straumann n!ce, and GC Initial (*p* > 0.05). The highest decrease in flexural strength after aging was found in the universal composite (40 s polymerization) (*p* < 0.001) and Vita Enamic (*p* < 0.001), while the lowest decrease was in the hybrid ceramics, Cerasmart and Lava Ultimate (*p* < 0.05). Extending polymerization duration reduced the aging effect on the universal composite tested. Thermomechanical aging affected the flexural strength of most materials tested. Universal composites and feldspar ceramics presented similar flexural strength values.

## 1. Introduction

A variety of CAD/CAM materials are readily available on the market, and using computer-aided design and computer-aided manufacturing (CAD/CAM) decreased clinical visits through faster manufacturing routes [1,2,3,4,5].

Ceramics and resin-based composites are the two main classes of dental restorative materials [6]. Ceramics are inorganic and composed of a crystalline phase and a glass matrix [7] and can be classified into three types: feldspar ceramics, glass ceramics (lithium disilicate, leucite), and polycrystalline ceramics (zirconia). Polycrystalline ceramics are stronger and tougher than feldspar and glass ceramics. Since they have a high crystalline content, they are more opaque than glassy matrix and felspar ceramics [8]. Manufacturers introduced infiltrated glassy matrix ceramics with additional particles in order to increase the strength. One example is lithium silicate, infiltrated with zirconia particles to reinforce the ceramic structure [9].

Resin-based composites consist of an organic polymer matrix and inorganic filler particles [10,11], with the organic part influencing dimensional changes and the amount of filler particle affecting Young’s modulus and hardness [11].

Ceramic restorations offer aesthetic benefits, high wear resistance, and strength [12,13,14], but are rigid and prone to fracture [15,16]. Direct resin composites, on the other hand, offer the advantages of less tissue removal, lower costs, and less abrasiveness, but may cause premature enamel cracking due to temporary flexural stresses caused by polymerization shrinkage [17]. On the other hand, hybrid ceramics combine the positive characteristics of ceramics and composite resins into a single material, forming two groups: polymer-infiltrated ceramic network (PICN) materials and resin nanoceramic (RNC) materials [8,18]. Their performance after aging conditions in the oral environment dictates the successful use of such materials for clinical indications.

Thermocycling, a method to mimic harsh oral environments [19,20], involves temperature, dwell time, and a number of cycles. Aging causes mechanical stresses, crack formation, and propagation in resins [21,22]. Alternating temperatures between 5 °C and 55 °C with 10–15 s dwell times are closest to oral cavity physiology, where 100,000 cycles were anticipated to be equivalent to one year of masticatory forces [19,20]. Modern CAD/CAM materials are regularly introduced to the market, necessitating a continuous comparison of their physical characteristics to ensure effective clinical application. Introduction of such new materials necessitates frequent comparison with and without aging conditions in order to determine the most effective and secure use of those materials. Preclinical investigations also reduce the need for costly RCTs and exposing patients to materials which perform inferiorly by comparison.

Therefore, the purpose of this study was to investigate and compare the values of flexural strength before and after thermomechanical aging for nine CAD/CAM restoratives and one universal resin composite with long and short polymerization durations. The long curing time of 120 s for the material Filtek Supreme XT was chosen to refine the composite, as it is suggested by the manufacturer with the CAD/CAM-composite Lava Ultimate.

The null hypotheses tested were that (a) thermomechanical aging would not reduce the flexural strength of the tested materials significantly and (b) ceramics would not present significantly higher flexural strength values than the universal composite and hybrid ceramics.

## 2. Material and Methods

### 2.1. Materials

**Figure 1:**
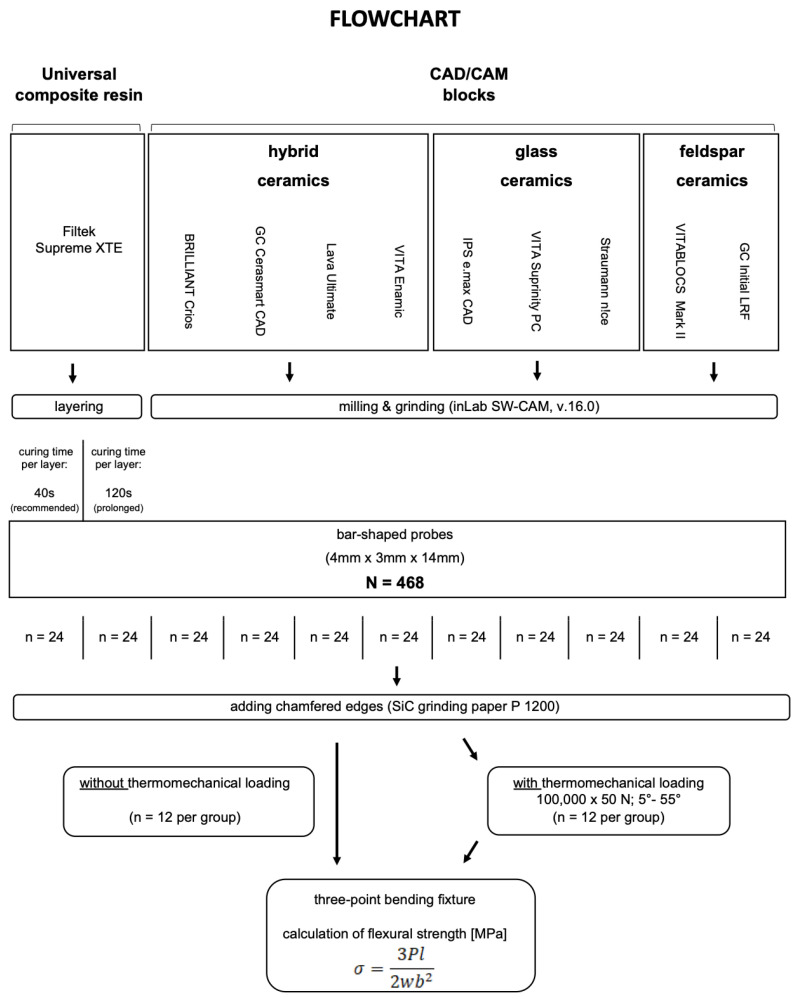
Flowchart of the experimental design.

The brands, types, manufacturers, and chemical compositions of all materials used in this study are listed in Table 1. The distribution of experimental groups and the experimental workflow are presented in Figure 1. In this study, 10 different materials (N = 264, *n* = 12 per group), of which one is a universal nanocomposite (a) and nine are CAD/CAM materials (b–h), were examined, namely (a) nanofiller composite (Filtek Supreme XTE-**XTE**, 3M ESPE, Seefeld, Germany), (b) resin nanoceramic (BRILLIANT Crios-**BRI,** Coltène/Whaledent, Altstätten, Switzerland), (c) resin nanoceramic (GC Cerasmart-**CER,** GC EUROPE N.V., Leuven, Belgium), (d) resin nanoceramic (Lava Ultimate-**LAV**, 3M ESPE, Seefeld, Germany), (e) polymer-infiltrated ceramic network (PICN) (VITA ENAMIC-**ENA**, VITA Zahnfabrik, Bad Säckingen, Germany), (d) lithium disilicate (IPS e.max CAD-**IPS**, Ivoclar Vivadent AG, Schaan, Liechtenstein), (e) lithium disilicate reinforced with zirconia (VITA Suprinity PC-**SUP**, VITA Zahnfabrik, Bad Säckingen, Germany), (f) lithium disilicate reinforced with aluminia (Straumann n!ce-**STR**, Straumann AG, Basel, Switzerland), (g) feldspar ceramic (VITABLOCS Mark II-**MAR**, VITA Zahnfabrik, Bad Säckingen, Germany), and (h) feldspar ceramic reinforced with leucite (GC Initial LRF-**LRF**, GC EUROPE N.V., Leuven, Belgium).

**XT** (a) was manufactured into a bar shape (3 × 4 × 14 mm^3^). The manufacturer recommends layering with a maximal increment depth of 1.5 mm for a polymerization duration of 40 s per increment, and an intensity of at least 400 mW/cm^2^ in the 400–500 nm wavelength range (dentin shade A1–D). Every specimen was composed of six layers. Each layer had a triangular shape and was photopolymerized in a laboratory polymerization device (Spectramat, Ivoclar Vivadent AG) either for 40 s (**XT1**) or for 120 s (**XT2**). After layering, all edges were chamfered with a 1200-grit silicon carbide grinding paper (WS flex 18 C, Hermes Schleifmittel GmbH, Hamburg, Germany).

CAD/CAM materials (b-h) were also fabricated into a bar shape (3 × 4 × 14 mm^3^). First, specimens were designed digitally (inLab SW-CAM Software, v.16.0, Dentsply Sirona Inc., York, PA, USA). Then, milling and grinding were conducted according to the manufacturer’s instructions with a wet milling and grinding unit (inLab MC XL, Dentsply Sirona Inc., York, PA, USA) using burs (Step Bur 12S and Cylinder Pointed Bur 12S, Dentsply Sirona Inc.). Attachment points were smoothed with a polisher (SD 716F.150 HP, Jota Kit 1433, JOTA AG, Rüthi, Switzerland). A chamfer was added to every probe with a 1200-grit silicon carbide grinding paper (WS flex 18 C, Hermes Schleifmittel GmbH, Hamburg, Germany). The quality of the bars was then controlled under a digital microscope (Keyence VHX 200, Osaka, Japan) with a magnification of ×200 in order to identify the presence of defects, cracks, or other imperfections within the material. No specimens with macroscopic defects or anomalies were identified during this investigation.

Afterwards, according to the manufacturer’s guidelines, **IPS** (Programat CS4, Ivoclar Vivadent AG) and **SUP** were sintered (Vacumat 6000 M, VITA Zahnfabrik H. Rauter GmbH, Neumarkt am Wallersee, Austria).

Half of all specimens were thermomechanically aged in a custom-made chewing simulator (University of Zurich, Clinic of Conservative and Preventive Dentistry) (A) with a 49 N force and a 1.67 Hz loading frequency. Simultaneously, the surrounding water temperature was changed between 5 and 55 °C every 120 s. The other half (**NA**) of the specimens were not subjected to any thermomechanical aging process.

All specimens were then tested in a three-point bending fixture (Z010, ZwickRoell GmbH & Co, Ulm, Germany) where the specimens were mounted flat on two support rollers with a center-to-center distance of 12 mm (the same probe placing design as in the thermomechanical loading machine) and loaded to fracture. For every specimen, the breaking load [N] and the exact dimensions (mm) were noted. Three-point flexural strength values (MPa) were calculated by the formula described in ISO standard No 6872:2008 [23] (E) as follows:

$$\sigma =\frac{3Pl}{2wb^{2}}$$

where *P* is the breaking load (N), *l* is the test span (mm) (center-to-center distance between support rollers), *w* is the width of the specimen (mm) (the dimension of the side at right angles to the direction of the applied load), and *b* is the thickness of the specimen (mm) (the dimension of the side parallel to the direction of the applied load).

### 2.2. Statistical Analysis

Mean flexural strength values (MPa) were calculated (Excel v16 Microsoft, Washington, USA). Statistical analysis was conducted (SPSS Software V.25, Chicago, IL, USA) with a two-sided significance level (α = 0.05) and a sample size of 12 in each experimental group. A sample size of 10 in each group was calculated to be sufficient to show significant differences when a drop in flexural strength value of 30% occurs.

Two statistical tests were applied to the mean values of each group. A Wilcoxon rank-sum test was conducted to measure the effect of aging. The means of each material group that did undergo aging (**A**) were compared with the same material groups that did not undergo aging (**NA**). The Kruskal–Wallis test (non-parametric test), followed by a Conover post hoc test, was adjusted after Holm for multiple testing to analyze the effect of the material on the results. For this statistical test, groups were split into subgroups **A** (with aging) and **NA** (no aging). The means of each experimental group were compared with the means of all other experimental groups in the same subgroup (**A** or **NA**). *p* values less than 0.05 were considered to be statistically significant in all tests.

## 3. Results

**Figure 2:**
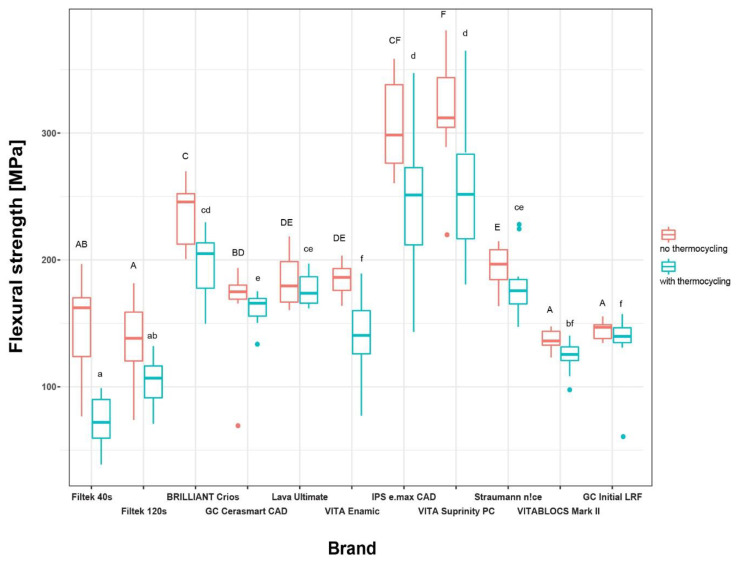
Boxplots representing the mean flexural strength values [MPa] for the materials tested. Same uppercase and lowercase letters indicate no significant difference for non-aged and aged groups, respectively.

Most of the tested materials (XT1, XT2, BRI, CER, ENA, IPS, SUP, and MAR) showed a significant decrease in flexural strength value (MPa) after aging compared to no aging when both mean and median values were considered (Table 2 and Table 3). The highest decrease in flexural strength occurred for XT1 (*p* < 0.001) and for ENA (*p* < 0.001). Aging caused no significant reduction in flexural strength for LAV, STR, and LRF (*p* > 0.05). There were also significant differences in flexural strength values between the material groups in subgroup A and subgroup NA (Figure 2, Table 4 and Table 5).

In the non-aged group, SUP-NA (317.5 ± 40.9), IPS-NA (306.6 ± 35.9), and BRI-NA (235.7 ± 26) showed significantly higher values among all materials (*p* < 0.05). The difference between SUP-NA and IPS-NA as well as IPS-NA and BRI-NA was not significant, while the difference between SUP-NA and BRI-NA was statistically significant (*p* = 0.048). STR-NA (193.9 ± 18.1) showed significantly lower strength values than all other lithium-reinforced ceramics, SUP-NA and IPS-NA, and also than the hybrid ceramic BRI-NA. BRI-NA showed significantly higher strength values than all other hybrid ceramics (CER-NA, LAV-NA, and ENA-NA). Lithium disilicate ceramics (IPS-NA, SUP-NA, and STR-NA) and hybrid ceramics (BRI-NA, CER-NA, LAV-NA, and ENA-NA) had higher strength values than both resin composites (XT1-NA, XT2-NA) and feldspar ceramic materials (MAR-NA, LRF-NA). The mean flexural strength values of resin composites (XT1-NA, XT2-NA) and feldspar ceramics (MAR-NA, LRF-NA) were similar (*p* > 0.05) while XT1-NA and XT2-NA also presented similar results compared to each other (*p* > 0.05) (Table 4).

In the aged group, the mean flexural strength values of SUP-A (253.4 ± 51.5) and IPS-A (246.31 ± 62.39 MPa) were significantly higher than the values of all other tested materials. The strength of BRI-A (197.15 ± 23.48 MPa) was not significantly higher than that of STR-A (180.06 ± 24.14 MPa) (*p* > 0.05). There was no difference between STR-A, BRI-A, and LAV-A (*p* > 0.05). Except for ENA-A, all lithium disilicate and hybrid ceramics had significantly higher strength values than composite and feldspar ceramic materials (*p* < 0.001). ENA-A showed a lower strength value than all other hybrid ceramics (*p* < 0.05). While LRF-A and MAR-A presented similar results (*p* > 0.05), LRF-A (feldspar ceramic) had significantly higher strength values than both resin composite groups (XT1-A, *p* < 0.001, and XT2-A, *p* = 0.008). There was no difference between XT1 and XT2 (*p* > 0.05) (Table 5).

## 4. Discussion

The objective of this study was to evaluate the three-point flexural strength of one universal nanocomposite material and nine different CAD/CAM hybrid, glass, and feldspar ceramic materials before and after thermomechanical aging. The bars created for the strength test measured 3 × 4 × 14 mm^3^, which corresponds to a three-unit restoration in clinical settings. The results of flexural strength testing showed that three CAD/CAM materials did not decrease in strength significantly after aging conditions, namely the resin nanoceramic “RNC” Lava Ultimate, the aluminum-reinforced lithium silicate Straumann N!ce, and the leucite-reinforced feldspar ceramic GC Initial LRF, while all other tested materials showed a significant decrease in strength. The highest decrease was observed for the regularly polymerized nanocomposite Filtek Supreme XTE (−51%) and the polymer-infiltrated nanoceramic “PICN” VITA Enamic (−25%). Hence, the first hypothesis was partially rejected.

The universal nanocomposite that we tested presented comparable results to feldspar ceramics before thermocycling. However, the universal nanocomposite demonstrated the lowest strength values of all tested materials after the aging process. All glass ceramics and hybrid ceramics showed higher mechanical stability than feldspar ceramics and the universal nanocomposite. Therefore, the second hypothesis was also partially rejected.

Flexural strength tests are crucial for assessing the mechanical performance of dental restorative materials, as they are susceptible to failure due to repetitive occlusal loading [24,25]. Flexural strength testing provides accurate comparisons of the 10 materials due to standardization, testing method, and the controlled laboratory environment [26]. The three-point bending test is a common, reliable, and efficient screening method for assessing the strength of particularly brittle materials such as ceramics and composites. The test serves to evaluate the performance of the materials which undergo bending stresses in service, while also excluding the possible confounding factors such as occlusal anatomy, height, and the connector diameter of FDPs. Additional tests could be performed to include anatomic parameters with the best-performing materials after the three-point bending test.

Thermomechanical aging is the best aging method for simulating clinical situations but standardized protocols are still lacking [20]. In most studies, the number of cycles is not well described and chosen randomly, with some suggesting 1,200,000 cycles for 5 years in vivo [27] while others suggest 10,000 cycles for 1 year of clinical function [21]. Although standardized guidelines for testing are still required, in this study, 100,000 cycles were performed to observe the early effects of aging on the materials tested, which should correspond to at least 1 year and up to 10 years of clinical service [28].

The results of the study suggest that the clinical success of resin-based restorations may be impacted by insufficient polymerization [29]. Longer polymerization times can increase the degree of polymerization [30]. However, this study found that regular polymerization for a duration of 40 s per increment led to a higher decrease in strength (−51%) than prolonged polymerization of 120 s (−23%). Therefore, the authors recommend prolonged polymerization time for the resin composite Filtek Supreme XTE, especially when the material is used in stress-bearing areas, such as its use for three-unit fiber-reinforced resin-bonded fixed dental prostheses that are directly built in the oral cavity.

This study found that resin nanoceramics (RNCs) showed higher flexural strength than polymer-infiltrated network (PICN) hybrid ceramics, confirming previous findings on hybrid ceramics [18,31,32]. One previous study found no significant differences in the strength of PICN and RNC hybrid ceramics after 400,000 cycles of thermomechanical aging conditions [3]. Manufacturers reported a flexural strength of 246 MPa for GC Cerasmart, which is 45.8% higher than the mean value obtained in this study. Factors such as specimen dimensions, testing conditions, and test type can explain these differences [33]. This study also reveals a significant decline in strength after the aging of the polymer-infiltrated network “PICN” VITA Enamic, possibly due to different compositional and microstructural complexities in each material group [18] where the size and morphology of filler particles in hybrid ceramic materials, water uptake, and mechanical properties could influence their flexural strength [34]. Nonetheless, the percentage decrease in strength was similar for both hybrid ceramics and glass ceramics.

The mechanical properties of dental glass ceramics are linked to their crystal structure, with crystalline particles increasing fracture strength [6]. This could explain their higher flexural strength compared to other materials [35]. VITA Suprinity PC, IPS e.max CAD, and Straumann N!ce have all presented lower flexural strength values than what was reported by the manufacturers. According to the results, the mean strength values of IPS e.max CAD and VITA Suprinity PC were on the other hand identical but decreased following thermomechanical aging, suggesting that the addition of zirconia reinforcement to lithium silicate had minimally beneficial effects on flexural strength. When compared to Straumann N!ce, IPS e.max CAD and Vita Suprinity PC exhibited noticeably greater strength both before and after thermal cycling. Unlike Straumann N!ce, the latter two require additional firing for final crystallization following milling, which could help reduce the defects created during the manufacturing process [3,36].

Previous studies showed no statistical decrease in the flexural strength of glass and feldspar ceramic materials after thermomechanical aging [37,38,39]. However, the flexural strength of zirconia-reinforced ceramics and lithium disilicate ceramics in the present study significantly decreased after 100,000 cycles. These results are in agreement with those of a former study [40].

The results of this study indicated no significant decrease in strength for the leucite-reinforced feldspar ceramic GC Initial LRF after aging, suggesting that the reinforcement of the feldspar glassy matrix with leucite positively affects its durability. According to the results, CAD/CAM lithium disilicate ceramics show the highest mechanical properties, while leucite ceramics show the lowest, which is in accordance with a previous study [41].

Although flexural strength is a relevant mechanical indicator, dental ceramics also need to withstand the chemical and thermal conditions in the oral cavity. The results of this study may be beneficial for conducting randomized clinical trials to analyze the clinical survival of different types of CAD/CAM materials. This study suggests that indirect restorations (CAD/CAM) are superior for anterior multi-unit FDPs, while feldspar ceramics and PICN hybrid ceramics cannot be recommended due to a high percentage decrease in strength after aging which may be detrimental in their clinical durability. The authors also advise prolonged polymerization time for the use of universal nanocomposite materials used for direct FDPs.

The mechanical properties of CAD/CAM materials are not well understood, especially for the novel hybrid ceramic material class and the zirconia-reinforced leucite ceramic GC Initial LRF. This study calls for standardized thermomechanical aging procedures to compare the results and consider the exposure of the materials to prolonged aging conditions to determine the best-performing materials. The prismatic specimens do not reflect the clinical conditions in the case of FDPs, which could be considered as a limitation of three-point flexural strength tests. Also, in minimally invasive applications, reduced thickness of the tested materials may lead to lower flexural strength results [42]. Therefore, the results should be interpreted considering the dimensions of the specimens in such tests. Furthermore, the possible presence of defects, cracks, or other imperfections within the material could be investigated using scanning electron microscopy images to assess the quality of the bars, provided that ideal settings during milling and regular changes of the milling units reduce such flaws in the specimen or the restoration. Future studies should investigate the mechanical durability of the tested materials when bonded onto enamel and dentin as the adhesion properties may alter the mechanical strength of both the resin composite and the CAD/CAM materials.

## 5. Conclusions

From this study, the following could be concluded:

1. The flexural strength of the nano-filled resin composite and polymer-infiltrated ceramic network materials were more affected by the thermomechanical aging.
2. Glass ceramics and hybrid ceramics presented higher flexural strength values than feldspar ceramics and universal composites regardless of thermomechanical aging, where the latter two presented similar flexural strength results. The lack of significant strength reduction in glass ceramics and hybrid ceramics after aging suggests their potential viability for clinical use in stress-bearing applications.
3. The prolonged polymerization duration from 40 to 120 s per increment improved the mechanical durability of the universal nanocomposite material. During fabrication of direct FDPs, clinicians should consider polymerization durations longer than 120 s.
4. Given their inferior performance against aging, universal composite and feldspar ceramics should be used with caution in cases of extensive dental reconstructions in load-bearing areas.
5. All CAD/CAM materials should be exposed to artificial aging prior to clinical application as they showed reduction in their mechanical strength at varying degrees. The results of this study need to be verified when they are bonded to enamel and dentin as adhesive properties may affect their mechanical strength.

## Figures and Tables

**Table 1:** Classifications, brands, structural and chemical compositions, manufacturers, and batch numbers of the materials tested.

Material Class	Brand	Structural Composition (All), Crystallization (Only Ceramics)	Chemical Composition (as Detailed as Available)	Manufacturer	Properties and Ref.-Nr.
**Universal resin** composite	**Filtek Supreme XTE**	Nanofiller composite	Organic part: UDMA and Bis-EMA with small amounts of TEGDMA and PEGDMA Inorganic part: 78.5 wt% fillers of SiO_2_ and ZrO_2_	3M ESPE, Seefeld, Switzerland	A1-D (4910A1D)
**Hybrid ceramic** (CAD/CAM block)	**BRILLIANT Crios**	RNC (Resin Nano Ceramic) with dispersed fillers	Organic part: Bis-GMA, Bis-EMA, TEGDMA (cross-linked methacrylates) Inorganic part: 70.7 wt% fillers of barium glass and SiO_2_	Coltène/Whaledent, Altstätten, Austria	A1 HT 14 (60019994)
	**GC Cerasmart**		Organic part: Bis-MEPP, UDMA, DMA Inorganic part: 71 wt% fillers of barium glass and SiO_2_	GC EUROPE N.V., Leuven, Belgium	A1-HT-14 (008507)
	**Lava Ultimate**		Organic part: Bis-GMA, UDMA, Bis-EMA, TEGDMA Inorganic part: 80 wt% SiO_2_ and ZrO_2_ fillers and aggregated clusters	3M ESPE, Seefeld, Switzerland	14L, A1-HT (3314A1HT)
	**VITA ENAMIC**	PICN (Polymer-Infiltrated Ceramic Network) with a polymer-infiltrated ceramic network	Organic part: UDMA, TEGDMA Inorganic part: glass ceramic sintered network (SiO_2_, AlO_2_, Na_2_O, K_2_O, B_2_O_3_, ZrO_2_, CaO)	VITA Zahnfabrik, Bad Säckingen, Germany	1M1-HT, EM-14 (EC41M1HTEM14)
**Glass ceramic** (CAD/CAM block)	**IPS e.max CAD**	Lithium disilicate glass ceramic (*partially crystallized*)	Lithium disilicate (LiSi_2_) crystalline particles embedded in a glass matrix	Ivoclar Vivadent AG, Schaan, Liechtenstein	A1-HT-C14 (626407)
	**VITA Suprinity PC**	Lithium disilicate glass ceramic infiltrated with zirconia (*partially crystallized*)	Zirconia (ZrO_2_) crystalline particles embedded in a glass matrix; SiO_2_, Li_2_O, ZrO_2_	VITA Zahnfabrik, Bad Säckingen, Germany	A1-HT, PC-14 (EC4S010130)
	**Straumann n!ce**	Lithium disilicate glass ceramic infiltrated with lithium aluminosilicate (fully crystallized)	Lithium disilicate (LiSi_2_) crystalline particles embedded in a glass matrix; SiO_2_, Li_2_O, AL_2_O_3_	Straumann AG, Basel, Switzerlnad	A1-HT-14L (010.7042)
**Feldspar ceramic** (CAD/CAM block)	**VITABLOCS Mark II**	Feldspar ceramic (fully crystallized)	Feldspathic crystalline particles embedded in a glass matrix	VITA Zahnfabrik, Bad Säckingen, Germany	A1C-HT, I-14 (EC4A1CI14)
	**GC Initial LRF**	Feldspar ceramic infiltrated with leucite (fully crystallized)	Pigments are frit with feldpar before mixing in the ceramics; SiO_2_, AL_2_O_3_, K_2_O, Na_2_O, Li_2_O	GC EUROPE N.V., Leuven, Belgium	A1-HT-14 (876502)

Organic components: DMA: dimethacrylate; UDMA: urethane dimethacrylate; Bis-EMA: ethoxylated bisphenol-A dimethacrylate; Bis-GMA: bisphenol-A diglycidylether methacrylate; TEGDMA: triethylene glycol dimethacrylate; PEGDMA: Polyethylene glycol dimethacrylate; Bis-MEPP: Bisphenol-A-ethoxylat dimethacrylat; Inorganic components: SiO_2_: Silicium-dioxide («Silica»); AL_2_O_3_: Aluminum-oxide («Alumina»); ZrO_2_: Zirconium-dioxide («Zirconia»); Na_2_O: Natrium-oxide; B_2_O_3_: Bor-trioxide; K_2_O: Potassium-oxide; Li_2_O: Lithium-oxide.

**Table 2:** Group abbreviations, brands, mean flexural strength values ± standard deviation, and median ± IQR (MPa) with (A) and without thermomechanical aging (NA) and reduction in percentage after aging. Same lowercase letters in the same row indicate no significant difference (*p* < 0.05).

Group	Brand	Number of Specimen	Flexural Strength [MPa] (Mean ± SD) (*n* = 12 per Group)		Reduction in Mean After Aging (%)	Flexural Strength [MPa] (Median ± IQR) (*n* = 12 per Group)		Reduction in Median After Aging (%)
			No Loading	With Loading		No Loading	With Loading	
XT1	Filtek (40 s curing)	12-A, 12-NA	148.45 ± 37.72 a	72.90 ± 19.83 b	−51%	162.25 ± 46.29	71.88 ± 30.49	−56%
XT2	Filtek (120 s curing)	12-A, 12-NA	136.10 ± 31.98 a	104.78 ± 18.47 b	−23%	138.23 ± 38.47	106.84 ± 25.23	−23%
BRI	BRILLIANT Crios	12-A, 12-NA	235.67 ± 25.99 a	197.15 ± 23.48 b	−16%	245.70 ± 39.85	204.84 ± 35.75	−17%
CER	GC Cerasmart CAD	12-A, 12-NA	168.65 ± 32.68 a	161.19 ± 11.65 b	−4%	174.76 ± 11.11	165.91 ± 13.85	−5%
LAV	Lava Ultimate	12-A, 12-NA	183.66 ± 20.46 a	176.86 ± 12.63 a	−4%	179.46 ± 31.93	173.76 ± 20.79	−3%
ENA	VITA Enamic	12-A, 12-NA	183.94 ± 13.11 a	137.26 ± 35.40 b	−25%	186.22 ± 17.28	140.47 ± 34.00	−25%
IPS	IPS e.max CAD	12-A, 12-NA	306.62 ± 35.90 a	246.31 ± 62.39 b	−20%	298.49 ± 61.87	251.16 ± 60.99	−16%
SUP	VITA Suprinity PC	12-A, 12-NA	317.51 ± 40.93 a	253.39 ± 51.45 b	−20%	312.03 ± 39.19	251.65 ± 66.66	−19%
STR	Straumann n!ce	12-A, 12-NA	193.93 ± 18.06 a	180.06 ± 24.14 a	−7%	196.64 ± 23.63	175.68 ± 19.19	−11%
MAR	VITABLOCS Mark II	12-A, 12-NA	137.03 ± 8.01 a	123.78 ± 11.82 b	−10%	136.06 ± 10.90	125.46 ± 10.73	−8%
LRF	GC Initial LRF	12-A, 12-NA	144.61 ± 7.39 a	135.79 ± 25.08 a	−6%	146.85 ± 10.82	139.72 ± 11.77	−5%

**Table 3:** *p*-values indicating significant differences between flexural strength values for materials with (A) and without aging (NA) (Wilcoxon rank-sum test).

Group	Brand	Aging (No-NA/Yes-A)	Flexural Strength (Mean ± SD) (*n* = 12 per Group)	*p*-Value (Wilcoxon Rank-Sum Test)
XT1	Filtek 40 s	NA	148.45 ± 37.72	<0.001
		A	72.90 ± 19.83	
XT2	Filtek 120 s	NA	136.10 ± 31.98	0.008
		A	104.78 ± 18.47	
BRI	BRILLIANT Crios	NA	235.67 ± 25.99	0.006
		A	197.15 ± 23.48	
CER	GC Cerasmart CAD	NA	168.65 ± 32.68	0.017
		A	161.19 ± 11.65	
LAV	Lava Ultimate	NA	183.66 ± 20.46	not significant
		A	176.86 ± 12.63	
ENA	VITA Enamic	NA	183.94 ± 13.11	<0.001
		A	137.26 ± 35.40	
IPS	IPS e.max CAD	NA	306.62 ± 35.90	0.006
		A	246.31 ± 62.39	
SUP	VITA Suprinity PC	NA	317.51 ± 40.93	0.002
		A	253.39 ± 51.45	
STR	Straumann n!ce	NA	193.93 ± 18.06	not significant
		A	180.06 ± 24.14	
MAR	VITABLOCS Mark II	NA	137.03 ± 8.01	0.004
		A	123.78 ± 11.82	
LRF	GC Initial LRF	NA	144.61 ± 7.39	not significant
		A	135.79 ± 25.08	

**Table 4:** *p*-values indicating significant differences between flexural strength values for materials without aging (NA) (Wilcoxon rank-sum test).

No Aging (NA)	XT1	XT2	BRI	CER	LAV	ENA	IPS	SUP	STR	MAR	LRF
XT1 (Filtek Supreme XTE—40 s)	-	n.s.	<0.001	n.s.	<0.001	<0.001	<0.001	<0.001	<0.001	n.s.	n.s.
XT2 (Filtek Supreme XTE—120 s)	n.s.	-	<0.001	<0.001	<0.001	<0.001	<0.001	<0.001	<0.001	n.s.	n.s.
BRI (BRILLIANT Crios)	<0.001	<0.001	-	<0.001	<0.001	<0.001	n.s.	0.048	0.036	<0.001	<0.001
CER (GC Cerasmart)	n.s.	<0.001	<0.001	-	n.s.	n.s.	<0.001	<0.001	0.048	<0.001	<0.001
LAV (Lava Ultimate)	<0.001	<0.001	<0.001	n.s.	-	n.s.	<0.001	<0.001	n.s.	<0.001	<0.001
ENA (VITA ENAMIC)	<0.001	<0.001	<0.001	n.s.	n.s.	-	<0.001	<0.001	n.s.	<0.001	<0.001
IPS (IPS e.max CAD)	<0.001	<0.001	n.s.	<0.001	<0.001	<0.001	-	n.s.	<0.001	<0.001	<0.001
SUP (VITA Suprinity PC)	<0.001	<0.001	0.048	<0.001	<0.001	<0.001	n.s.	-	<0.001	<0.001	<0.001
STR (Straumann n!ce)	<0.001	<0.001	0.036	0.048	n.s.	n.s.	<0.001	<0.001	-	<0.001	<0.001
MAR (VITABLOCS Mark II)	n.s.	n.s.	<0.001	<0.001	<0.001	<0.001	<0.001	<0.001	<0.001	-	n.s.
LRF (GC Initial LRF)	n.s.	n.s.	<0.001	<0.001	<0.001	<0.001	<0.001	<0.001	<0.001	n.s.	-

n.s.: not significant.

**Table 5:** *p*-values indicating significant differences between flexural strength values for materials with aging (A) (Kruskal–Wallis test).

With Aging (A)	XT1	XT2	BRI	CER	LAV	ENA	IPS	SUP	STR	MAR	LRF
XT1 (Filtek Supreme XTE—40 s)	-	n.s.	<0.001	n.s.	<0.001	<0.001	<0.001	<0.001	<0.001	0.008	<0.001
XT2 (Filtek Supreme XTE—120 s)	n.s.	-	<0.001	<0.001	<0.001	<0.001	<0.001	<0.001	<0.001	n.s.	0.008
BRI (BRILLIANT Crios)	<0.001	<0.001	-	<0.001	n.s.	<0.001	n.s.	n.s.	n.s.	<0.001	<0.001
CER (GC Cerasmart)	<0.001	<0.001	<0.001	-	n.s.	0.049	<0.001	<0.001	n.s.	<0.001	0.009
LAV (Lava Ultimate)	<0.001	<0.001	n.s.	n.s.	-	<0.001	0.003	<0.001	n.s.	<0.001	<0.001
ENA (VITA ENAMIC)	<0.001	0.001	<0.001	0.049	<0.001	-	<0.001	<0.001	<0.001	0.156	n.s.
IPS (IPS e.max CAD)	<0.001	<0.001	n.s.	<0.001	0.003	<0.001	-	n.s.	0.004	<0.001	<0.001
SUP (VITA Suprinity PC)	<0.001	<0.001	n.s.	<0.001	<0.001	<0.001	n.s.	-	<0.001	<0.001	<0.001
STR (Straumann n!ce)	<0.001	<0.001	n.s.	n.s.	n.s.	<0.001	0.004	<0.001	-	<0.001	<0.001
MAR (VITABLOCS Mark II)	0.008	n.s.	<0.001	<0.001	<0.001	0.156	<0.001	<0.001	<0.001	-	n.s.
LRF (GC Initial LRF)	<0.001	0.008	<0.001	0.009	<0.001	n.s.	<0.001	<0.001	<0.001	n.s.	-

n.s.: not significant.

## Data Availability

The original contributions presented in the study are included in the article, further inquiries can be directed to the corresponding author.
